# Supplementary material for: The Construction and Exploration of a Comprehensive MicroRNA Centered Regulatory Network in Foxtail Millet (Setaria italica L.)
Source: Front Plant Sci. 2022 May 6;13:848474. doi: 10.3389/fpls.2022.848474 (PMC9121102; doi:10.3389/fpls.2022.848474)
Supplement: Supplementary file 3 [file Data_Sheet_3.zip › Sit-miR169f_Seita.9G129400.1_2100_TPlot.pdf]

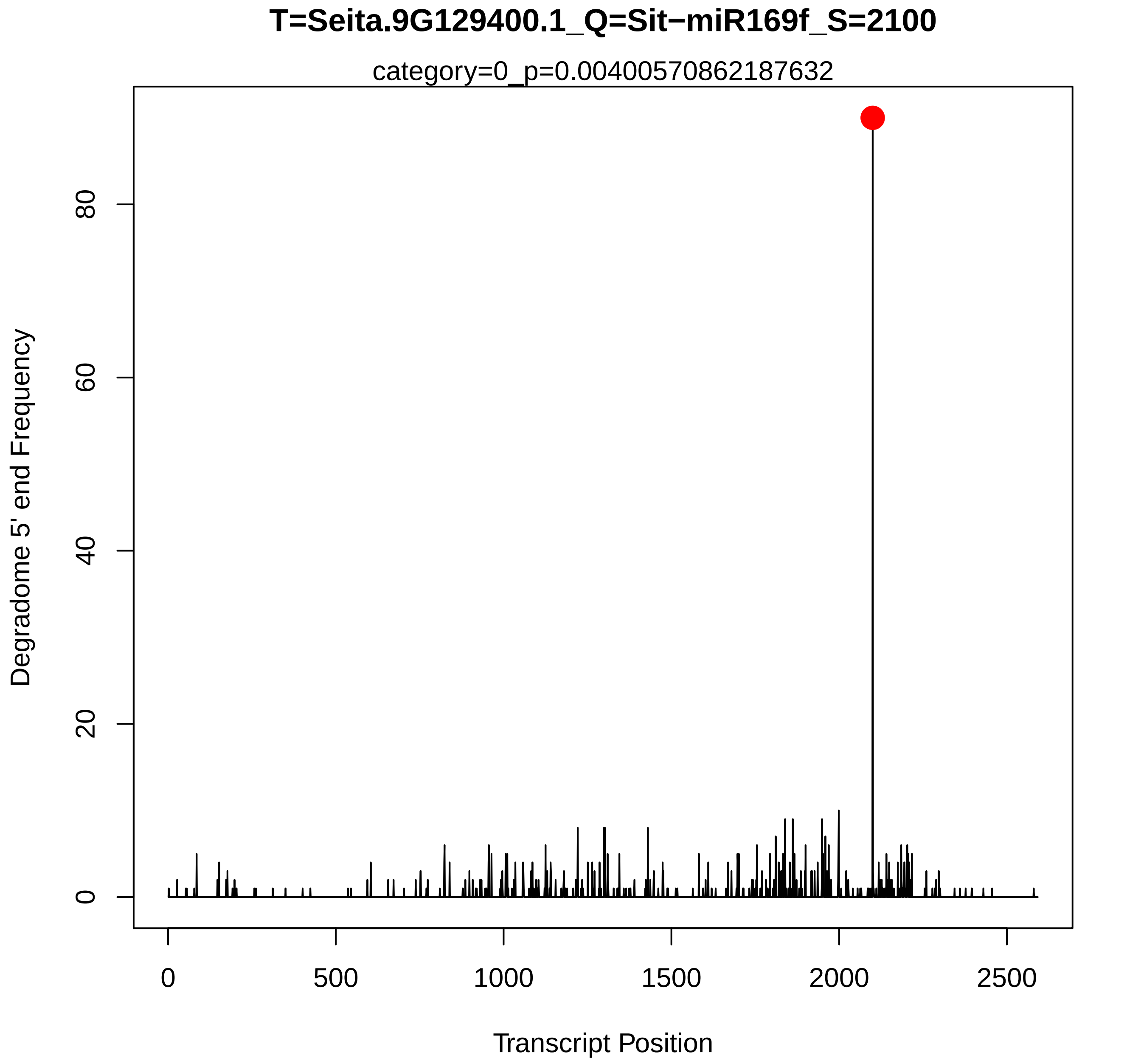
T=Seita.9G129400.1_Q=Sit−miR169f_S=2100
category=0_p=0.00400570862187632
Degradome 5' end Frequency
0
20
40
60
80
0
500
1000
1500
2000
2500
Transcript Position
